# Supplementary material for: Validation of Knock-Out Caco-2 TC7 Cells as Models of Enterocytes of Patients with Familial Genetic Hypobetalipoproteinemias
Source: Nutrients. 2023 Jan 18;15(3):505. doi: 10.3390/nu15030505 (PMC9921550; doi:10.3390/nu15030505)
Supplement: Supplementary file 1 [file nutrients-15-00505-s001.zip › nutrients-2127106-supplementary.pdf]

nutrients

MDPI

Article

Validation of Knock-Out Caco-2 TC7 Cells as Models of Enterocytes of Patients with Familial Genetic Hypobetalipoproteinemias

Claire Bordat [1,2], Donato Vairo [1], Charlotte Cuerq [2,3], Charlotte Halimi [1], Franck Peiretti [1], Armelle Penhoat [2], Aurélie Vieille-Marchiset [2], Teresa Gonzalez [1], Marie-Caroline Michalski [2], Marion Nowicki [1], Noël Peretti [2,4,*] and Emmanuelle Reboul [1,*]

1 Aix-Marseille Université, INRAE, INSERM, C2VN, 13885 Marseille, France
2 CarMeN Laboratory, INSERM U1060, INRAE, UMR 1397, Université Claude Bernard Lyon 1, 69495 Pierre-Benite, France
3 Biochemistry Department, Hospices Civils de Lyon, 69495 Pierre-Benite, France
4 Pediatric Hepato-Gastroenterology and Nutrition Unit, Hôpital Femme Mère Enfant (HFME) de Lyon, Hospices Civils de Lyon, 69677 Bron, France
* Correspondence: noel.peretti@chu-lyon.fr (N.P.); emmanuelle.reboul@univ-amu.fr (E.R.)

Supplementary Data

Table S1. DNA sequences targeted by guide-RNA used in CRISPR/Cas9 experiment.

Assessed Function	Gene Name	Gene ID	Target DNA sequence (5′ – 3′)
**Genes of interest involved in chylomicrons formation**	*MTTP*	4547	ACGCTCCTTCATCTAATCCA
	SAR1B	338	TTGACTCTAACAGCCTTTCG
**Positive control, housekeeping gene**	*HPRT1*	3251	GCATTTCTCAGTCCTAAACA

Table S2. Genomic primers used in CRIPSR/Cas9 experiment and for gene amplification prior to Sanger sequencing.

Gene Name	Gene ID	Forward Primer	Reverse Primer
MTTP	4547	GCAGAGAGGAGAGAAGAGCA	CCAAGGCTGAGGCTGTCAGA
SAR1B	338	GCTGGGACTACAGACCCT	CTGAATGCCCGACCCTTAGA
HPRT1	3251	ACATCAGCAGCTGTTCTG	GGCTGAAAGGAGAGAACT

Table S3. Primer sequences used in off-target experiments.

Complete Name	Gene Name	Gene ID	Forward Primer	Reverse Primer
Bromodomain and PHD finger containing 1	*BRPF1*	7862	GGGAGGGAAATCTCAACCATG	TAATCCACAGCACGAGAGGGT
Interleukin 34	*IL34*	146433	AGCTTTCATCCCCCTCTGTTG	TGACTTCCACGGTAAGGGCT
Mannosidase Alpha Class 1A Member 1	*MAN1A1*	4121	CAAGCATGGAGAAGGCAGTT	CCCCCTCAACCATTCAATCTC
MCF.2 cell line derived transforming sequence like	*MCF2L*	23263	CAAAACAAAACCCAAACCCAC	ACATCCCACACTCAGGAAG
Tenascin XB	*TNXB*	7148	CCTTTCCGAGGGTTCAGTCC	CCTCTTTCCGCCTCTCACAG
Cysteine Sulfinic Acid Decarboxylase	*CSAD*	51380	AAATGAAGAAGCGGGAAGGGT	AAAAGAGAGCATCCACGCCT
Signal peptide, CUB Domain and EGF like Domain Containing 3	*SCUBE3*	222663	ACAGGAAGACGTGCAAAGGT	CCAGTACAGCCTTTCGAGGG

Table S4. Primer sequences used in RT-qPCR experiments.

Assessed Function	Gene Name	Gene ID	Forward Primer	Reverse Primer
**Fatty acid transport**	*SLC27A4*	10999	GCTTCATCCGGGTCTTCATC	AGTTGGCTACACTGCTTGAG
	FABP2	2169	TAGCAGACGGAACTGAACTC	GTGCGCCAAGAATAATGCTC
**Chylomicron formation**	*APOB*	338	GCCATGTCCAGGTATGAGCTC	TTCTCCGGGTAAAGGAAAACC
	MTTP	4547	TGTTCAAGACATCCTACGTTTGGA	TGAGCGACCATTTCCTTCAGA
	SAR1A	56681	TCCAACACTACATCCGACATCA	TTTCCAAACGCGACGTGC
	SAR1B	338	ATTGCTGGCATGACGTTTAC	TGCCATTGATAGCAGGAAGG
**Cholesterol metabolism**	*ABCA1*	19	ACAACATGAATGCCATTTTCCA	ATAATCCCCTGAACCCAAGGA
	ABCG8	64241	CACTCGCAGCCCTGTTTCTA	TTTCCACACAGGTGTCCTCG
	NPC1L1	2881	GGCAGACCTCCCAAGTCGA	ATCCTTGAAGGTGAGCGGG
	PGP	283871	ATCAGCAGCCCACATCATCA	TCCTTCCAATGTGTTCGGCA
	SCARB1	949	AGTCTGGAATTCAGAACGTC	GTGGATGTCCAGGAACAAGG
**Intracellular lipid metabolism**	*ACAT2*	39	AGGTGAGATGCCACTGACTG	TTCTGGGACAGAACTGCAAC
	DGAT1	8694	CAGTGGCTTCAGCAACTACC	CAGAGAAACCACCTGGATGG

	DGAT2	84649	AAGGGCTTTGTGAAACTGGC	CCTCCTCGAAGATCACCTGC
	MGAT2	80168	CCCTCCCACTCTTTCATGGC	GATGGGCTTCCCCACCAC
	PLIN2	123	ATCCAAGATCAAGCCAAGCA	AAGGGACCTACCAGCCAGTT
**Oxidative stress management**	*CAT*	847	CTGTGAACTGTCCCTACCGT	AATTTGGAGCACCACCCTGAT
	SOD1	6647	CTAGCGAGTTATGGCGACGAA	CACTGGTCCATTACTTTCCTTCTG
